# 3D Printable Device for Automated Operant Conditioning in the Mouse

**DOI:** 10.1523/ENEURO.0502-19.2020

**Published:** 2020-04-27

**Authors:** Raffaele Mazziotti, Giulia Sagona, Leonardo Lupori, Virginia Martini, Tommaso Pizzorusso

**Affiliations:** 1 Department of Neuroscience, Psychology, Drug Research and Child Health (NEUROFARBA), University of Florence, Florence 50135, Italy; 2 Institute of Neuroscience, National Research Council, Pisa 56124, Italy; 3 Department of Developmental Neuroscience, Istituti di Ricovero e Cura a Carattere Scientifico (IRCCS) Stella Maris Foundation, Pisa 56128, Italy; 4 BIO@SNS Laboratory, Scuola Normale Superiore, Pisa 56124, Italy

**Keywords:** 3D printing, Arduino, conditioning, learning, Raspberry Pi, reaction times

## Abstract

Operant conditioning (OC) is a classical paradigm and a standard technique used in experimental psychology in which animals learn to perform an action to achieve a reward. By using this paradigm, it is possible to extract learning curves and measure accurately reaction times (RTs). Both these measurements are proxy of cognitive capabilities and can be used to evaluate the effectiveness of therapeutic interventions in mouse models of disease. Here, we describe a fully 3D printable device that is able to perform OC on freely moving mice, while performing real-time tracking of the animal position. We successfully trained six mice, showing stereotyped learning curves that are highly reproducible across mice and reaching >70% of accuracy after 2 d of conditioning. Different products for OC are commercially available, though most of them do not provide customizable features and are relatively expensive. This data demonstrate that this system is a valuable alternative to available state-of-the-art commercial devices, representing a good balance between performance, cost, and versatility in its use.

## Visual Abstract

**Figure:**
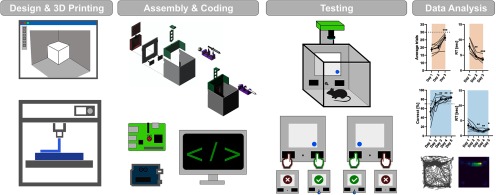

## Significance Statement

3D printing is a revolutionary technology that combines cost-effectiveness with an optimal trade-off between standardization and customization. Here, we show a device that performs operant conditioning (OC) in mice using largely 3D-printed parts. This tool can be employed to test learning and memory in models of disease. We expect that the open design of the chamber will be useful for scientific teaching and research as well as for further improvements from the open hardware community.

## Introduction

Operant conditioning (OC; Jones et al., 1939) is a standard technique used in experimental psychology in which animals, like rodents (Francis and Kanold, 2017; O'Leary et al., 2018), reptiles (Mueller-Paul et al., 2014), birds (Cook, 1992), dogs (Range et al., 2008), monkeys (Range et al., 2008), and humans (Siqueland, 1964; Angulo-Barroso et al., 2017), learn to perform an action to achieve a reward. By using this paradigm, it is possible to extract learning curves and measure accurately mental chronometry [e.g., reaction times (RTs)]. As previously suggested (Escobar and Pérez-Herrera, 2015; Francis and Kanold, 2017; O'Leary et al., 2018), different products for OC are commercially available, though most of them do not provide customizable features and are relatively expensive. Neuroscience research has greatly benefited from new 3D printing technologies bringing new possibilities to build tools, and increasing productivity and user timeliness. 3D printing also opened unprecedented resources for training students and solving common experimental problems (Baden et al., 2015). There is a plethora of work using 3D-printed mechanical parts (Baden et al., 2015), ranging from fluorescence microscopes (Chagas et al., 2017) to electrophysiology systems (Siegle et al., 2017). The combination of 3D printing with off-the-shelf, low-cost optical and electronic components facilitates reproducibility of experimental tools internationally and promotes rapid iteration and prototyping (Chagas et al., 2017). Here, we demonstrate an affordable, fully 3D printable, and automated solution that can be reproduced rigorously in any laboratory equipped with a 3D printer with a total cost around 160 € (Table 1). We designed the chamber entirely using 3D modeling for several reasons: first, it has a high degree of reproducibility, since the model is standardized and can be downloaded to print the same structure with the same materials throughout different laboratories. Second, it can be easily customized in relation with specific experimental needs. Lastly, it can be easily shared through on-line repositories. With these cost-efficient and accessible components, we assayed the possibility to perform two-alternative forced choice OC using audiovisual cues while tracking in real time mouse position.

**Table 1:** Bill of materials

Material	Price (€)	Vendor	Code	Manufacturer
LED MATRIX	26.74	amazon.it	B071VJL91V	Kuman:WS01
Stepper motor	3.38	amazon.it	B00DGNO6PI	Elegoo
PLA	16.66	amazon.it	B06W568X1G	TECHNOLOGY OUTLET
Pi camera	18.99	amazon.it	B07P8PG5MF	Bewinner: Bewinnertyv48w6mf5
Raspberry PI	44.51	amazon.it	B01CD5VC92	raspberrypi
Graphene PLA	27.50	filoprint.it	PLA_GRAFENE_175	Haydale
Arduino UNO	16.85	amazon.it	B07SL2W4CL	Arduino: A000066
Power supply	5.69	amazon.it	B00UVOHJ0Y	Samsung:TA10EWE
Piezo Buzzer	1.35	adafruit.com	PS1240	tdk
Cables/wires	2.00	Off the shelf		
Total	163.67			

## Materials and Methods

### Mice housing and handling

Animals were kept at a constant temperature (22°C) with a standard 12/12 h light/dark cycle (7 A.M. to 7 P.M.). Food was available *ad libitum* and changed weekly. During OC protocol, mice are water restricted (body weight >85%; Goltstein et al., 2018) of their baseline. Before the experiment mice are handled for 1 h/d for one week. After the last daily session, mice had free access to water for 1 h (23 h of water deprivation). All the experiments were conducted in accordance with the directives of European Community Council (2011/63/EU) and approved by the Italian Ministry of Health. We tested six wild-type C57BL/6J [from postnatal day (P)50 to P180, four female and two male mice; Charles River].

### 3D-printed OC chamber

**Figure 1.**
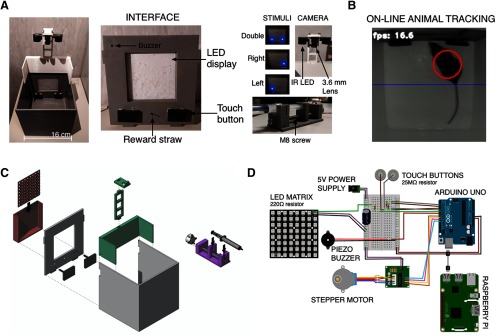
3D printable OC apparatus. ***A***, left, Top-view of the apparatus. Center, Interface wall. Right, Blue dotted stimuli, camera holder, and syringe pump. ***B***, An animal during the task, the blue line delimitates the “active zone.” ***C***, An exploded view of the project showing the assembling scheme. ***D***, Circuit diagram of all the components.

The OC arena (16 × 16 × 16 cm; thickness, 3 mm; Fig. 1*A*) is 3D printed using gray or white PLA (B06W568X1G, Technology Outlet). The 3D project is designed using FreeCAD software, exported in stereolithography (STL) format, converted to G-code using Cura (https://ultimaker.com/software/ultimaker-cura) and printed using Kentstrapper Verve 3D printer (https://kentstrapper.com/stampante-3d-kentstrapper-verve/). In Figure 1*C*, an exploded-view drawing of the chamber is shown. The color coding corresponds to different components of the apparatus (visual stimulation parts in red; camera holder in green; syringe pump in purple). All these components are coated using epoxy transparent resin (LF-L2GR-26GX, resinpro), which allows cleaning (5% ethanol in water). The arena front wall contains the elements interfacing the animal with the computer. It can be modularly assembled to the arena and is composed by a squared frame containing the LED matrix at the center, four holes for the touch buttons in the lower part, a central hole for the lick spout and a hole in the upper part to connect a piezo buzzer. The touch buttons are printed using graphene PLA (PLA_GRAFENE_175, filoprint), and connected using conductive glue (Chemtronics: CW2400) to a female pin (B07XQHD752, amazon.it) using a resistor (25 MΩ). A dotted grid is interposed between the LED matrix and the inside of the chamber and has two roles: first, the dotted pattern restricts the visibility of the LED lights to equal small circles; second, it contains a grid of walls facing the LEDs that prevents the light from each source to spill over to the neighboring dots. The LED matrix is covered with a thin white Plexiglas foil, so that single LED are not visible if they are off and to diffuse light uniformly. The camera holder, is joint assembled on top of the frontal wall and it is designed to maintain the camera at the distance necessary to image the entire arena using a 3.6-mm focal length objective. The syringe pump is composed of a base that fixes the barrel of the syringe into position and of a piston that slides on a stepper motor guided M8 metal screw and allows to push or pull the plunger.

### Hardware

An electronic board is mounted on a grounded metal sheet and is composed by a Raspberry Pi connected via USB to an Arduino UNO (AU) board (https://store.arduino.cc/arduino-uno-rev3). The Raspberry Pi (https://www.raspberrypi.org/products/raspberry-pi-3-model-b/) acts as the main computer of the setup. It executes the Python three script that handles the structure of the experiment, performs computer vision using a Raspberry PI camera (Bewinner: Bewinnertyv48w6mf5), and saves data (Fig. 1*B*). The AU controls sensors and actuators in the OC chamber. Two touch buttons, made using conductive PLA, acts as capacitive sensors and are connected to AU using coaxial cables (3 mm in diameter) to minimize environmental noise. The main advantage of using graphene PLA resides in the possibility to print different button designs (e.g., for motor impairment, nose poking, etc.). There are three actuators: a LED matrix serves as display (Adafruit: 1487), a piezo buzzer (Adafruit: PS1240, frequency range: 2–10 kHz, 60 dB) is used as acoustic stimulator glued at the top of the frontal door, and a stepper motor (amazon.it: 28BYJ-48, with ULN2003) connected to a M8 screw guiding the piston of a syringe pump controlling a disposable syringe (10 ml) connected with a silicone tube equipped by luer tapers adapters to a blunt needle (Warner Instruments: SN-18) for reward delivery. This modular configuration allows the proper cleaning of the delivery tubing after each session. We use an external 5-V 2-A DC power supply (Samsung: TA10EWE) with a 1000-μF capacitor to power the LED matrix and the stepper motor. A diagram of the electrical wiring is shown in the Figure 1*D*.

### Software

#### AU program

The code controlling the OC box is organized in four files, the file called *skinner.ino* contains the logic of the experiment and manages the serial communication with the computer. Different files are dedicated to different aspects of the program: the file called *button.ino* contains functions to control the touch buttons and play auditory stimuli, the file called *ledLib.ino* contains wrapper functions to control Adafruit NeoPixel library (https://www.adafruit.com/product/1487) and generate simple visual stimuli easily, the third file called *stepper.ino*, contains functions to control the syringe pump using the Arduino Stepper motor library (https://www.arduino.cc/en/reference/stepper). In summary, to setup the AU, a user needs to download the folder containing the .*ino* files, uncompress and upload the file *skinner.ino*.

#### Raspberry Pi program

**Figure 2.**
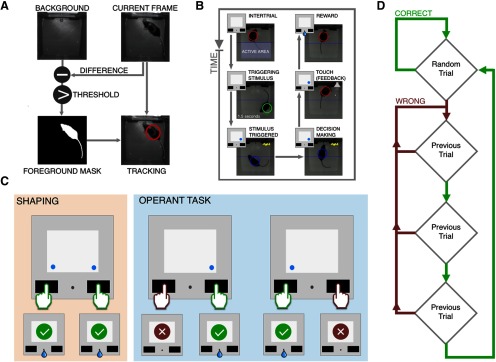
Behavioral procedures. ***A***, The detection of the mouse is obtained using background subtraction from the current frame and then applying a threshold, isolating only the mouse silhouette. ***B***, Behavioral sequence to obtain the reward. ***C***, Diagram showing the behavioral procedures, during the shaping phase and the operating task. ***D***, Flowchart of the assisted procedure.

On the Raspberry Pi, a Python script controlling the experiment has been written using IDLE. The program relies on a number of external libraries that are required to run all parts of the script with no errors. Since the task relies on real time tracking of the animal position we use *picamera* and *opencv* libraries to acquire frames and process them using K-nearest neighbors based background-foreground segmentation (Zivkovic and van der Heijden, 2006), a widely used algorithm for generating a foreground mask using static cameras (Fig. 2*A*). The technique consists of two main steps, the first one is the background initialization in which we use 1000 frames of the empty arena, then we set the learning rate to zero and the algorithm stops updating the background so it is ready to locate reliably the position of the animal with a frame rate of 20 Hz. *LibSerial* library is used to communicate with the AU during the task sending symbolic codes and changing the state of the AU in the OC chamber. We used *Tkinter* library to write the initial GUI to set the experimental parameters. The behavioral sequence is outlined in Figure 2*B*. Virtually the chamber can be divided into two sections: the anterior part that contains the interface between the mouse and the computer, and the posterior side that is designed as an active area to activate the trials. If the mouse remains in the active area for a given amount of time (1.5 s) the trial is triggered. At this stage a visual stimulus is shown on the display and the system waits for animal response. When the mouse touches one of the two buttons, an auditory feedback is produced, with a tone that varies depending on whether the answer is correct (3300 Hz) or wrong (2700 Hz). In case of correct answer, a drop (7 μl) of water with 1% condensed milk is released.

### Implementation of an LCD screen

As a proof of principle of customizability, we added a version of the OC chamber that is able to show more complex visual stimuli. This version includes an edit of the frontal wall that can host a TFT monitor (Kookye 3.5” for RPI3) and a folder (LCD_oc_chamber) containing code that runs on Psychopy2 (Peirce, 2008), a Python package dedicated to behavioral experiments. This configuration allows to show RGB images as visual stimuli.

### Code accessibility

The code described in the paper is freely available online at https://github.com/raffaelemazziotti/oc_chamber. The code is also available as Extended Data 1.

10.1523/ENEURO.0502-19.2020.ed1 Extended Data 1 zip contains the code for both Arduino and Raspberry Pi boards. Download Extended Data 1, ZIP file.

### The OC protocol

#### Familiarization

This phase is conducted by placing each animal in the OC box for three sessions of 10 min, spaced by at least 2 h between each other. During this phase, a liquid reward, coupled with the “correct” tone is provided manually whenever the mouse is in the active area, in this way the animal learns where to find the reward and associate it with the tone.

#### Shaping (3 d)

The visual stimulus is introduced (Fig. 2*C*). It consists of two bright (0.9 cd/m^2^) blue (465–475 nm) dots (5 mm) that appear above the two buttons. The mouse needs to touch one of the two buttons to obtain the reward.

#### Operant task (OT, 5 d)

During this phase, only one dot appears, identifying the correct button. If the mouse touches the correct button, the correct tone is reproduced and the animal receives the reward. If the mouse touches the wrong button, the “wrong” tone is reproduced and no liquid reward will be delivered. This procedure is shown in Figure 2*C*. The sequence of stimuli is balanced so that the mouse sees each case the same number of times. In order to prevent perseveration with the same answer, during the first 2 d we adopted an assisted procedure (Fig. 2*D*): the first stimulus presented is random, if the mouse produces the correct answer the following stimulus is randomized, in case of wrong answer instead, the system repeats the same stimulus until the mouse gives the correct answer three times.

#### Follow-ups

In order to test the ability of the mouse to recall the task, we tested animals in different follow-ups, respectively, at 6 d, 27 d, three months, and four months approximately after the end of OT. For each recall sessions we tested mice once per day.

### Data analysis and statistics

Data processing is performed using Python and the statistical analysis (Table 2) using GraphPad Prism 7. To analyze mouse tracking data the arena is virtually divided into 256 (16 × 16) bins and raw exploration is z-scored to obtain relative exploration measures. To quantitatively test whether the mouse preferentially explores some of the bins, we constructed a resampled binned exploration matrix representing chance level for each bin, randomly permuting each animal exploration matrix for 100 times. The software Fritzing was used to draw the wiring diagram of the electrical components. We used Rhino 6 to draw the exploded version of the model.

**Table 2:** Statistical table

Figure	Type of test	Statistical data
Fig. 3*A*, average trials	RM one-way ANOVA, Dunnett’s multiple comparisons *post hoc*	*p* = 0.0006, *post hoc* day 1 vs day 3, *p* < 0.001
Fig. 3*A*, RT	As above	*p* = 0.0002, day 1 vs day 2, *p* = 0.001 and day 1 vs day 3, *p* = 0.0002
Fig. 3*A*, ITI	As above	*p* = 0.0002, day 1 vs day 2, *p* = 0.0148 and day 1 vs day 3, *p* = 0.0001
Fig. 3*C*, average trials	As above	*p* = 0.0046, day 1 vs day 3, *p* = 0.0057; day 1 vs day 4, *p* = 0.0057 and day 1 vs day 5, *p* = 0.0036
Fig. 3*C*, RT	As above	*p* = 0.0022, day 1 vs day 3, *p* = 0.0027; day 1 vs day 4, *p* = 0.0011 and day 1 vs day 5, *p* = 0.0275
Fig. 3*C*, ITI	As above	*p* < 0.0001, day 1 vs day 3, *p* = 0.0004; day 1 vs day 4, *p* < 0.0001 and day 1 vs day 5, *p* = 0.0002
Fig. 3*C*, correct	As above	*p* = 0.0025, day 1 vs day 2, *p* = 0.0464; day 1 vs day 3, *p* = 0.0043; day 1 vs day 4, *p* = 0.0042 and day 1 vs day 5, *p* = 0.0013
Fig. 3*D*, average trials	As above	*p* = 0.0058; baseline vs 4 months, *p* = 0.0042
Fig. 3*D*, RT	As above	*p* < 0.0001, baseline vs 6 d, *p* = 0.0063; baseline vs 4 months, *p* < 0.0001
Fig. 3*D*, ITI	As above	*p* < 0.0001, baseline vs 27 d, *p* = 0.0093, baseline vs 3 months, *p* = 0.0009, baseline vs 4 months, *p* = 0.0252
Fig. 3*D*, correct	As above	*p* = 0.2290
Fig. 4*B*, relative exploration	As above	*p* < 0.0001, corner vs center, *p* < 0.0001; corner vs reward, *p* < 0.0001, center vs reward, *p* < 0.0001
Fig. 4*C*, correlation matrix TR vs RT	Spearman’s correlation	*r* = –0.8223 (95% CI, –0.9335 to –0.5669); *R*^2^ = 0.6761
Fig. 4*C*, correlation matrix TR vs ITI	Spearman’s correlation	*r* = –0.9472 (95% CI, –0.9811 to –0.8573); *R*^2^ = 0.8971
Fig. 4*C*, correlation matrix ITI vs RT	Spearman’s correlation	*r* = 0.9209 (95% CI, 0.7910 to 0.9714); *R*^2^ = 0.8480
Fig. 4*C*, correlation matrix DIST vs RT	Spearman’s correlation	*r* = 0.5666 (95% CI, 0.1208 to 0.8222); *R*^2^ = 0.3210
Fig. 4*C*, correlation matrix DIST vs ITI	Spearman’s correlation	*r* = 0.4972 (95% CI, 0.02450 to 0.7882); *R*^2^ = 0.2472
Fig. 4*D*, tracking distance	One-way ANOVA, Holm–Sidak’s *post hoc*	*p* = 0.03; day 1 vs day 3, *p* = 0.02

## Results

### Behavioral performance

**Figure 3.**
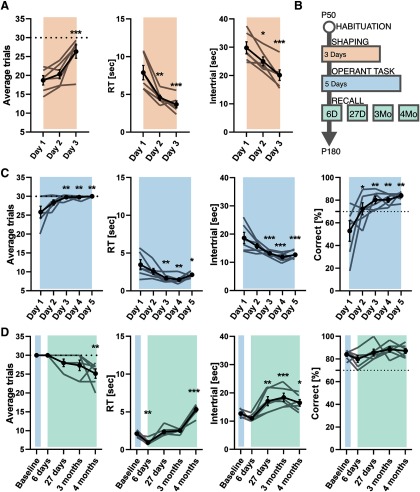
Behavioral performance. ***A***, Performance of the shaping phase. ***B***, OC protocol. ***C***, Performance during the OT. ***D***, Performance during recall.

To test our system ability to detect learning curves, we trained mice as depicted in the protocol in Figure 3*B*. Table 2 reports all statistical analysis. In shaping phase, the average number of trials (TR) progressively increases over time for all the subjects and specifically the third day, we detect a significant increase compared with the first day. Moreover, RTs and intertrial intervals (ITIs) showed a similar trend with a significant reduction of the RT starting from the second day (Fig. 3*A*). This indicates that already at day 2, the animals started to refine the sequence of actions necessary to trigger the stimulus and produce a response. Next, the results of the OT phase are shown in Figure 3*C*. The average TR continued to grow until day 3 of OT. After this day, the majority of the animals performed the maximum TR permitted in each session. Both RT and ITI showed a decrease with time. Indeed, RT and ITI dropped significantly during the first 2 d and reached a plateau by the third day. We observed a significant difference in the percentage of correct responses between the first and second day. In order to assess the retention of the test over time, we tested the same mice at different time points after the end of the OT. Accuracy remained stable during all time points tested; however, RT showed a more complex pattern: with an initial decrease, compared with the last day of the OT, followed by an increase at four months. Analyzing ITI, we detected an increase of the time between two trials at 27 d that remained higher at three and four months compared with the end of the OT (Fig. 3*D*). It is interesting to note that, since touch sensors are activated from all sides, some of the variability in timing performance could be explained by the development of different strategies to activate sensors.

### Tracking analysis

**Figure 4.**
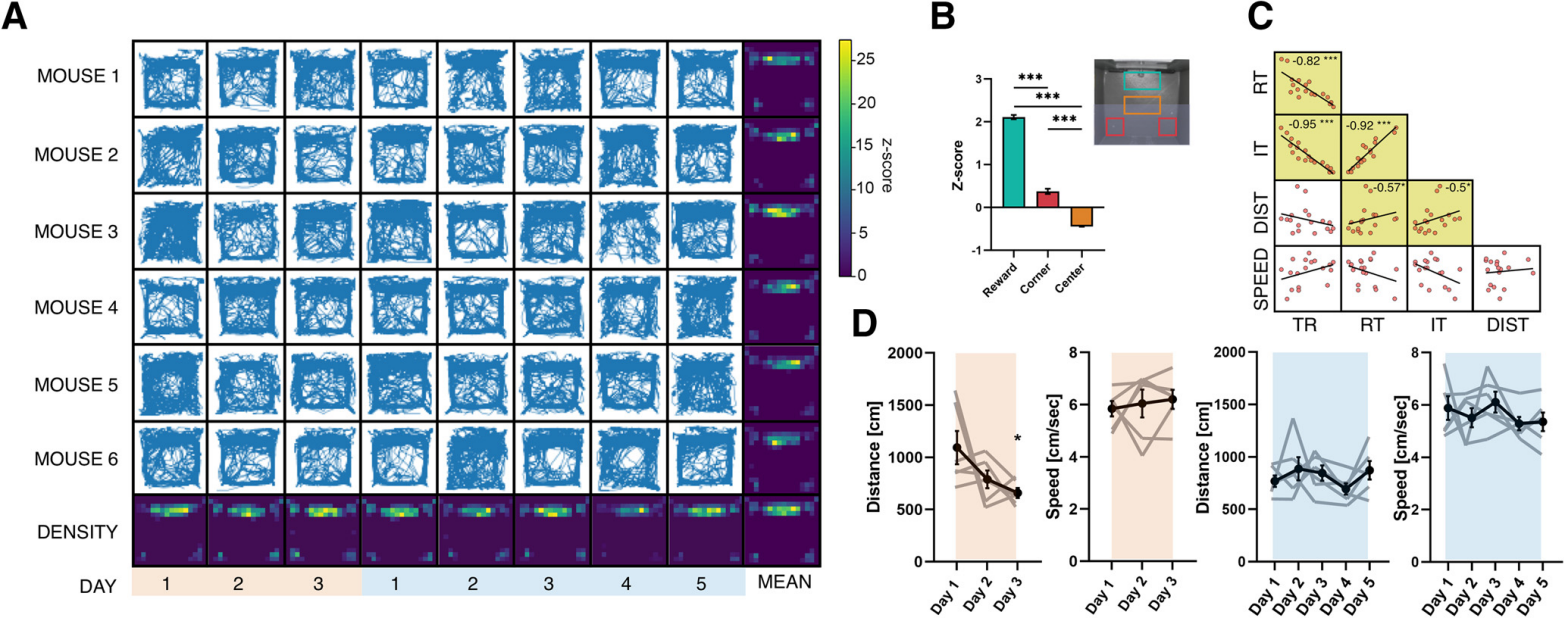
Tracking analysis. ***A***, Matrix of tracking traces of all animals per all days, with marginal heatmaps, showing spots of exploration significantly different from chance. Average heatmaps per each animal and per each day are presented in the last column and in the last row, respectively. ***B***, Relative exploration in the arena: reward area is the most frequently explored followed by corners of the active area and the central spot. ***C***, Correlation analysis between performance and spatial tracking. ***D***, Velocity and distance traveled during the shaping phase and the OT.

In Figure 4*A*, tracking traces, of all the mice, are shown with corresponding heatmaps, averaged across animals (on the right) or days (bottom row), showing non-uniform exploration of the OC chamber during tasks. Pixels that were not significantly explored compared with randomly resampled uniform exploration values (*p* > 0.05) were set to 0. The reward area was the most visited place, as shown by both the animals and session average heatmaps. In the bottom half of the arena there are two significant exploration spots at the corners, that indicate a stereotyped strategy to activate the trial (Fig. 4*B*). Moreover, we analyzed the distance traveled by each animal inside the OC box during all the tasks. We found that, throughout the course of the shaping phase, there was a significant decrease in the total amount of distance traveled compared with the first day (Fig. 4). Conversely, during the OT phase, we detected no changes (*p* = 0.3672). Interestingly, we found a significant correlation between timing performances and the total distance moved during shaping (Fig. 4*C*), this suggests that ∼25% (*r*^2^ = 0.247) of the improvement in timing performance is explained by a reduction in the distance traveled and the response of the animal. In addition, no differences in the average speed were detectable during both shaping and OT. These results imply that the reduction of the RT is due to the optimization of the psychomotor sequence in realizing the task rather than to a general increase of velocity of the animal.

## Discussion

Here, we described a fully 3D printable device that performs OC on freely moving mice while tracking the animal position in real time. We successfully trained 6 subjects, showing stereotyped learning curves that are highly reproducible across mice and reaching >70% of accuracy after 2 d of conditioning (Movie 1). This dataset demonstrate that this system is a valuable low-cost alternative to available state-of-the-art commercial devices, representing a good balance between performance, cost, and versatility. Performances detected by our system in three sessions per day (3.97 ± 0.11 trial/min with an accuracy of 84.1 ± 1.7%) are comparable with normative values detected in C57BL/6J and measured on an analogous two-alternative forced choice task performed once daily (Malkki et al., 2010). Although the LED display does not allow to design complex visual patterns required to perform image recognition and classification, visual stimulation is flexible enough to design simple tasks to test attention, learning, memory and other neuropsychological aspects of cognition (D’Ausilio, 2012; Escobar and Pérez-Herrera, 2015). The system is also easily customizable, as it is possible to add a LCD display (Movie 2) guided by extra Python libraries (e.g., Pygame or Psychopy). The overall cost of the chamber is around 160 €, but can be further substantially reduced using cheaper boards compared with AU and a Raspberry Pi. There are other low-cost alternatives for OC (Escobar and Pérez-Herrera, 2015; Francis and Kanold, 2017; O'Leary et al., 2018); however, the main strength of the present device is the high degree of reproducibility, since the model is standardized and can be downloaded to print the same structure with the same materials throughout different laboratories. Second, it can be customized in relation with specific experimental needs (e.g., very young animals). Lastly, different versions of the OC chamber can be tested and shared through on-line repositories, such as Thingiverse (https://www.thingiverse.com/) and NIH Print Exchange (https://3dprint.nih.gov/). Moreover, the OC chamber includes real time tracking of the mouse position, a feature that could be used as second phenotyping measure of anxiety or stereotyped behaviors. Additionally it allows to analyze other aspects of behavior, such as inhibitory control (Munakata et al., 2011). For example, by increasing the time required to trigger a trial, it is possible to measure impulsivity or reproduce neuropsychological tests used on humans like delayed gratification or stop signal tasks (Pinkston and Lamb, 2011; Furlong et al., 2016). It is also plausible to couple the procedure with physiological recordings in freely moving conditions such as imaging techniques (e.g., fiber photometry) and electrophysiology. Thanks to the general-purpose input/output ports (GPIO) of both AU and Raspberry Pi boards, high precision synchronization of physiological recordings with behavioral events is accurately integrated within experimental recording paradigms. The simplicity and modularity of the apparatus can be exploited as an educational tool to train students in 3D printing and coding. For these reasons, we expect that the open design of the OC chamber will be useful for teaching and research as well as for further design improvements from the international open hardware community.

Movie 1. A movie of a session with 30 trials during OT. 10.1523/ENEURO.0502-19.2020.video.1

Movie 2. A proof of principle of LCD screen functioning inside the OC box, under the same light conditions of stimulation. 10.1523/ENEURO.0502-19.2020.video.2

## Synthesis

Reviewing Editor: Siegrid Löwel, University of Goettingen Decisions are customarily a result of the Reviewing Editor and the peer reviewers coming together and discussing their recommendations until a consensus is reached. When revisions are invited, a fact-based synthesis statement explaining their decision and outlining what is needed to prepare a revision will be listed below. The following reviewer(s) agreed to reveal their identity: Nick Andrews, Janelle Pakan.

This manuscript describes a low-cost alternative using a 3D printer for a potentially useful operant conditioning tool but requires revision to highlight the full potential of the system and make clear the advancements to the field. The principle advantages of such a low-cost system include the ability for anyone with a 3D printer to produce the same device from the standard design for a fraction of the cost of the commercial suppliers and the ability to customize the design from the freely available programming, if all necessary details are provided (see below).

Both reviewers agree that this manuscript is of interest, because commercially available devices can be quite expensive. However, this specific design is quite simplistic and does not necessarily advance the scientific field in any meaningful way. We therefore suggest that the authors make the advancements to the field clearer. There are after all many other similar papers already describing these types of self-made systems. This one does seem to be quite easy to implement on the surface and the authors generally did a good job describing the 3D printing and component hardware. Adding more extensive functionality may help with the novelty here a bit. If the authors put in some time to make their code as user friendly as possible (which does not seem to be the case currently with the code we had access to (no access yet to the online repository for the code, which may contain more extensive instructions). Please provide access, and make the code very user friendly and polished through, otherwise no one will bother to even try to print it. And without usable open source code, it is just a 3D printed cube, which is not terribly difficult to design on one’s own. In short, this device does not significantly advance the field, but, with revision, it could provide a nice affordable and accessible tool for standardized experiments across labs.

Extended data: Movie 1 should remain as extended data: it is more of interest than of scientific necessity (although, for anyone actually trying to implement this system in their lab it would be vital to see it in action in this way. Extended data 2 (code) could remain extended data as long as the code is accessible via an online repository. Realistically, this is likely better as extended data, as the code will likely expand and be updated long after publication. We suggest to include some basic functionality of how to run the system code though (which should not change significantly over time as long as this is a currently working system) either in the main paper or along with extended data.

Software comments: The reviewers could not test the functionality of the system without the necessary device, but did investigate the code (extended data 2) from the perspective of a new user and tried to figure out where to start with the installation etc. The figures and Movie 1 are quite convincing, so we expect the system is working in their own lab, however, in order to claim that the authors are providing the full working system and not just the 3D printing instructions, they need to add a readme file - in the very least - to facilitate installation and utility for new users.

General Comments:

The authors have designed and developed a low-cost operant conditioning chamber, for simple two-choice conditioning experiments in mice, the large extent of which can be 3D printed and is run using open source hardware and software. The authors nicely demonstrate the results of using this chamber in a left/right touch task in response to a left/right visual cue. The figures are of high quality and the functionality in this respect is convincing. The developed system is, however, quite simple in its current functionality and would benefit from the addition of an LCD screen, as the authors themselves suggest, which would dramatically increase the versatility of potential experiments using this device and represent a more significant advance. Regardless of this enhancement, the open source code provided to run the system also needs to be made more user friendly if use of this system is to be adopted by other labs.

The challenge that most systems are not customizeable needs to be qualified more. The operant systems from the 2 or 3 main suppliers (Med Associates, Coulbourn, Lafayette) are very modular and the software is programmable so that users can make new programs. What cannot be modified are the size of the food chambers or the chamber itself for example, but there is quite a lot of flexibility for combining different stimuli etc.

As there are no screws that I can see, and the system is glued together, if one needs to change something that is broken or not optimal, does the whole device need to be printed again or are there modular parts?? How easy is it for the mice to chew the plastic over time? Related to this, what are running costs for the system in terms of the need to replace parts? For example what if the reward port blocks, can this be replaced separately from the main device? What about the known frequency to replace parts? Commercial systems are made of metal and are robust. How reliable is the syringe pump to deliver the same amount of reward each time?

The authors need to be more specific about the cost comparison - the phrase that it costs 150 € to make should be more explicit in the description in the text of what that includes - it obviously doesn’t include the printer or the computer needed to run the device - anything else? For someone without a 3D printer and no knowledge of how to program a printer are there companies with a 3D printer and if so what price would that be? Is the program compatible with Mac and PC?

I can see that video tracking can be a useful addition for welfare concerns to check the animals are ok following drug treatment for example but I’m struggling to see what other advantages it has (maybe in the situation where there is a depression of responding in the presence of a drug???).

Detailled comments:

Abstract:

Line 9: ‘Fully 3D printable’ is a bit misleading. There are many parts required to run the device that are not 3D printable - without these parts, the 3D box would be entirely non-functional. Here, I think just removing the word ‘fully’ would be sufficient. See also line 22 in significance statement ‘only 3D printed parts’ - this should be toned down, perhaps ‘largely’.

I would suggest removing the references to the price in the abstract and also the intro (i.e. 150 Euro). This is of course very changeable and may not be accurate over time, and is also not very internationally relevant. Referring to the device as a low-cost option is sufficient, and the prices can be kept in Table 1 as a reference for specifics.

Introduction:

Line 38/39, the citation here of the Escobar 2015 study in relation to a statement about commercially available and expensive systems is a bit misleading as this is a paper describing a custom made low-cost operant training device. Either insert something like ‘except see...’ before this citation, move the citation to after ‘do not provide customizable features’ (if this was the point to be made with this citation), or reference other studies supporting the assertion.

Methods:

In terms of methodology described:

Please state the number of hours (23) of water restriction explicitly in the methods. Also state when lights were on in the light cycle as well as the strain, sex, age and supplier of the mice. It should be stated for those that are not experts in the area that it is not common to test mice 3 times per day (because normally larger cohorts than 6 are used) so the learning curves may not be representative of those produced with daily training sessions.

For the design of the chamber, it would be better to have the port on the opposite wall to the reward spout. This more optimal to prevent the mice from simply moving sideways from the response window to the food chamber which is easier.

Is there a house light? Is there a removable tray for cleaning purposes? Mice do not like walking around in their own urine and faeces.

Please indicate the version of Python used.

Line 75: the authors list their specific printer (Kentstrapper Verve 3D printer), but it would be very useful to know what are the important properties/parameters for 3D printing this specific operant device. Clearly, this is only a cheap alternative if labs already have a 3D printer equipped, so it would be useful to know explicitly if the properties of their model would be compatible with the design. Here, I would suggest listing some key parameters that are necessary for printing success with any model printer (minimum dimensions, resolution, filaments; any limitations?).

Line 86: a small statement about why graphene is used as the capacitive touch sensor (and any suggestions on alternatives that do/do not work that may have been tested) would be useful.

Line 87: reference for conductive glue?

Line 84: camera reference? I assume Pi camera from table 1. Please include this here.

Line 96: what precision does the stepper motor plunger have for reward dispensing? What is the smallest/largest reward drop size, is this adjustable, etc.

Line 110: what are the properties of the acoustic stimulator (frequency range etc).

Line 141: what does a frame rate greater than 10Hz mean? 10 Hz is the minimum? What frame rate do the authors routinely use? What is the max the system (Pi camera, Pi interface) can handle?

Line 143: How is this GUI run/accessed with the available code (see also comments on extended data 2).

Results:

In the extended data (movie 1), it is perhaps difficult to tell from the top view, but it appears as though the mouse sometimes touches the button on the correct side (sometimes climbing on top of the device, for instance at 3:30 in movie 1) but this does not set off the sensor until the mouse touches the front of the panel (?). Could the authors comment on this design, the apparent behaviour, and the sensitivity of the touch device in this regard. If it is the case that the front is more sensitive than the top/sides, would it not be more advantageous to put the touch sensors recessed into the frame of the device so that only the front surface is exposed to the animal. It would seem that this would produce more of a one-to-one relationship towards touching the correct button and receiving the reward. As it is, the mouse likely has some extinction because it is touching the correct side button but in the ‘wrong way’ so that does not set off the sensor. Can the authors comment on this either as an option or as a experimental design consideration. This would also clearly affect the validity of reaction time measures (could the authors comment on this in regard to long-term RT measures; Line 206). If it is the case that it is difficult to see from the video, and the touch sensor is activated from all sides, then please state this explicitly in the methods description and comment on the fact that the animal can develop many different strategies to activate the sensor (i.e. climb up on, hit side of, hit front of, etc), reducing the stereotyped nature of the behaviour and potentially affecting RT measures.

As far as we can tell, the extended data 2 does not include a readme file. This should be standard and would help with installation as well as general description of the provided code and associated files. Additionally, without this, it is difficult to evaluate how user friendly the open source software is for this system. Either way, we would encourage the authors, either in a readme file in extended data 2 or in a supplementary information directly within the manuscript, to provide a small example of code needed to run the system. Such as a simplified demo bit of code to get users started with the basic functions. In ourexperience, without this users can very quickly become frustrated and will not pursue using the open source system.

Discussion:

We are missing a more detailed description of how the current results using this OC chamber relate to other standardized systems/publications.

Line 234: the authors state the results produced stereotyped learning curves that were reproducible across mice, which speaks to the internal validation of the OC chamber, but how do these learning curves relate to extensive previously published data on operant conditioning? This discussion would be important for external validation of this system.

It would also be helpful to expand on what the range of tasks is that can be performed with the current device being described.

Line 245-248: The Francis and Kanold, 2017 system is designed for mice, contrary to the authors claims that this system and others are designed for rats. This is also a highly automated system that can be used in a home cage without extensive researcher supervision. Further, what would be the rationale that the Escobar 2015 system could not be used with mice?

In relation to this, we would generally like to see a more extensive comparison with previous devices/literature including specific advantages (and disadvantages) of the proposed system to aid in evaluation of the needs for specific experiments before implementation. What precisely does this system add to push the field forward. For instance, the combination of realtime tracking and analysis of movement together with the system is of interest and could be better highlighted in this regard (including references to previous designs).

The authors mention that the LED display can be ‘easily’ functionally expanded using Pygame to allow for more complex visual displays. We think this would be a significant advantage. Although this would increase the cost of the system, including this as an ‘option’ in the code and as an example would increase the appeal for this system. As is, we are not sure of the widespread usefulness for a simple left/right light device. If this is as easy as the authors suggest, we would like to see it as an option from the start, at least as a backbone for implementation in the open source code. In general, this functionality would significantly add to the novelty of this OC chamber.

Line 255/256 and 260/261: if the authors would like to claim that this is a useful tool for training etc, then it is especially important to have well documented code (comments throughout the python code are generally few and some comments are not in English) and a clear readme file with installation instructions, demo code etc (see also point regarding extended data 2 above).

Table 1: the authors list vendors for products as amazon, but of course amazon is only the distributor not the manufacturer of these products. Please list the manufactures name as well as a manufactures’ product code where possible. In the future, amazon may stop supplying these brands and the information would be lost. In table 1, perhaps the authors could keep the amazon reference (depending on journal policy), but in the text and main reference for table 1 I think the manufactures’ data would be more useful.

Minor comments:

Line 247: typo ‘has’ not ‘have’

Line 248: typo ‘includes’; add s

Table 1: add (Euro) after Price heading

## Author Response

Dear Editor,

We thank you and the reviewers for the constructive comments and the synthesis. We have answered all the

points raised in the reviews and we hope that the manuscript is now ready for publication. A point-to-point

rebuttal follows.

Best regards,

Dr. Raffaele Mazziotti

POINT-TO-POINT

---------------------------------------------

Synthesis of Reviews:

Significance Statement Comments for Author (Required):

N/A

Comments on the Visual Abstract for Author (Required):

In the diagram that summarizes the method please draw the chamber as a cube - as it stands it looks a bit like a rhombus which is not the case and is also something that is typical of the Bussey-Sacsida system (though the narrow area is by the food port in the latter).

We re-draw the visual abstract according to the suggestion.

Synthesis Statement for Author (Required):

This manuscript describes a low-cost alternative using a 3D printer for a potentially useful operant conditioning tool but requires revision to highlight the full potential of the system and make clear the advancements to the field. The principle advantages of such a low-cost system include the ability for anyone with a 3D printer to produce the same device from the standard design for a fraction of the cost of the commercial suppliers and the ability to customize the design from the freely available programming, if all necessary details are provided (see below).

Both reviewers agree that this manuscript is of interest, because commercially available devices can be quite expensive. However, this specific design is quite simplistic and does not necessarily advance the scientific field in any meaningful way. We therefore suggest that the authors make the advancements to the field clearer. There are after all many other similar papers already describing these types of self-made systems. This one does seem to be quite easy to implement on the surface and the authors generally did a good job describing the 3D printing and component hardware. Adding more extensive functionality may help with the novelty here a bit.

If the authors put in some time to make their code as user friendly as possible (which does not seem to be the case currently with the code we had access to (no access yet to the online repository for the code, which may contain more extensive instructions). Please provide access, and make the code very user friendly and polished through, otherwise no one will bother to even try to print it. And without usable open source code, it is just a 3D printed cube, which is not terribly difficult to design on one’s own. In short, this device does not significantly advance the field, but, with revision, it could provide a nice affordable and accessible tool for standardized experiments across labs.

Extended data: Movie 1 should remain as extended data: it is more of interest than of scientific necessity (although, for anyone actually trying to implement this system in their lab it would be vital to see it in action in this way. Extended data 2 (code) could remain extended data as long as the code is accessible via an online repository. Realistically, this is likely better as extended data, as the code will likely expand and be updated long after publication. We suggest to include some basic functionality of how to run the system code though (which should not change significantly over time as long as this is a currently working system) either in the main paper or along with extended data.

We added a file in the extended data (readme.md) carefully explaining how to run the code. Moreover, we added comments in the code files.

Software comments: The reviewers could not test the functionality of the system without the necessary device, but did investigate the code (extended data 2) from the perspective of a new user and tried to figure out where to start with the installation etc. The figures and Movie 1 are quite convincing, so we expect the system is working in their own lab, however, in order to claim that the authors are providing the full working system and not just the 3D printing instructions, they need to add a readme file - in the very least - to facilitate installation and utility for new users.

We added a file in the extended data (readme.md) carefully explaining how to run the code. This file contains:

1. Instruction for 3D printing of parts

2. Instructions for assembly of the system

3. Required libraries

4. Installation of the code

5. Detailed description of the GUI

General Comments:

The authors have designed and developed a low-cost operant conditioning chamber, for simple two-choice conditioning experiments in mice, the large extent of which can be 3D printed and is run using open source hardware and software. The authors nicely demonstrate the results of using this chamber in a left/right touch task in response to a left/right visual cue. The figures are of high quality and the functionality in this respect is convincing. The developed system is, however, quite simple in its current functionality and would benefit from the addition of an LCD screen, as the authors themselves suggest, which would dramatically increase the versatility of potential experiments using this device and represent a more significant advance. Regardless of this enhancement, the open source code provided to run the system also needs to be made more user friendly if use of this system is to be adopted by other labs.

Two general points are raised here: 1) LCD screen addition and 2) making the system more user friendly.

Concerning 1): We implemented a backbone for functionality with a Raspberry Pi- compatible LCD screen (Kookye 3.5” for RPI3, ∼= 30€). We choose this model for two reasons: first, it is compatible with Raspberry Pi and, as a result, does not require the cost of an external PC to be added to the price of the setup; second, it is natively a touch screen display, and we think that developing a basic implementation for this device could help future users to expand the functionality of the OC chamber even more. We also included a video showing this version of the chamber in extended_data_3, we mentioned this version in the README.md file, and we included a 3D project for a version of the interface wall that fits this LCD screen.

Concerning 2): the addition of comments in the code files and of a readme file should make all the steps of system printing, assembly, installation, and use more user friendly.

The challenge that most systems are not customizable needs to be qualified more. The operant systems from the 2 or 3 main suppliers (Med Associates, Coulbourn, Lafayette) are very modular and the software is programmable so that users can make new programs. What cannot be modified are the size of the food chambers or the chamber itself for example, but there is quite a lot of flexibility for combining different stimuli etc.

We agree with this statement: the main suppliers offer a lot of options in their devices, most of them are of good quality, but this comes at a price. Basic versions are generally very rigid in their configurations and offer low degree customizability. There are also brilliant exceptions such as ENV-307A, but the cost of this device is incomparably higher than the 3D printed OC chamber. In addition, the main advantage of 3D printing is the possibility to share your models for free, boosting the reproducibility of experimental results and possibly rising a community of makers dedicated on appetitive conditioning and accelerating research through the promotion of collaboration through web tools such as OpenBehavior (http://openbehavior.com/). Moreover, software customizability degree differs greatly based on the company’s policy. In the case of the OC box, the degree of personalization is very wide so you can redesign entirely your own chamber sharing your version on public repositories.

We discussed these points more thoroughly in the Discussion section.

As there are no screws that I can see, and the system is glued together, if one needs to change something that is broken or not optimal, does the whole device need to be printed again or are there modular parts??

The system is mostly joint assembled, so it is relatively easy to change most of the components such as buttons and reward delivery, only the LED and camera holders are glued to the frontal wall, but depending on the amount of glue used they can be safely removed.

How easy is it for the mice to chew the plastic over time?

We have been using the OC chamber for one year and chewing was only detected once. For this reason, the possible damage derived from this behavior, albeit higher than using other materials such as metals, is negligible. On the other hand, it is very easy and cheap to reprint the damaged parts (see next point).

Related to this, what are running costs for the system in terms of the need to replace parts?

We have been using the device for one year, without the need for printing new parts.

Even if some of the parts might need a replacement due to wear and tear, the cost would be low. In fact, the entire weight of the OC chamber is about 200g, and the average cost of a medium quality filament is 20€ per Kilos, corresponding to a cost of raw materials of about 4€ to replace all the printed parts on the chamber.

For example what if the reward port blocks, can this be replaced separately from the main device?

Non-3D-printed parts are designed to be as easily replaceable as possible. The reward system fits disposable syringes and all the tubing is connected by Luers adapters. In our experience, with proper maintenance and cleaning procedures (passing 10mL of 20% ethanol followed by 10mL of distilled water through the tubing) the reward straw never blocked. However, this part is glued to the frontal wall, so replacing it might require to print another front wall part (see previous point for costs).

What about the known frequency to replace parts?

To our knowledge, there is no need to replace parts in more than one year of usage.

Commercial systems are made of metal and are robust. How reliable is the syringe pump to deliver the same amount of reward each time?

The reward drop is on average 7,05 μL with a standard error of 0,78 μL. Since the amount of water reward is proportional to the rotational movement of the stepper motor, this quantity can be modulated by editing a low-level parameter in the file “stepper.ino” to adjust for one’s experimental needs.

The authors need to be more specific about the cost comparison - the phrase that it costs 150 € to make should be more explicit in the description in the text of what that includes - it obviously doesn’t include the printer or the computer needed to run the device - anything else?

We agree on the need to be more specific on the cost breakdown. In particular, the total cost of 150€ does not include the 3D printer itself, however, despite its usefulness as a tool in the lab, one does not necessarily need to add the cost of an entire printer. It is possible to use external 3D printing services just to pay for the printing service. The computer, however, was in fact included in the price (see Table1).

The Raspberry Pi is a credit card-shaped computer able to run an entire operating system called Raspian and based on Linux.

Additional components not included in the cost are:

● cables and jumper wires (mandatory); ● a monitor and USB mouse and keyboard (for connecting to the Raspberry Pi. Alternatively, it is possible to use another external PC or laptop to run the experiment in headless mode by connecting remotely to the Raspberry Pi, for example using Virtual Network Computing (VNC) protocol, thus eliminating the need for those peripherals)

We added some clarification of this info in the bill of materials For someone without a 3D printer and no knowledge of how to program a printer are there companies with a 3D printer and if so what price would that be?

There are many companies that provide 3D printing as a service, for example, https://all3dp.com/ quotes printing all the parts of the OC chamber for about 350€. In our experience, however, 3D printing as a service is almost never worth the cost, unless one has some very specific needs like printing with non-standard materials or achieving a very high printing resolution that would require buying a high-end 3D printer.

Recent 3D printer models are cheap (a model suitable for printing this chamber starts from 250€) and very user-friendly. Usually 3D printing an object only requires downloading the .stl model (that we provide for our system), load it on the 3D printer software and start the print using the graphical interface of the software, without any need to learn any programming or 3D modeling skills.

Is the program compatible with Mac and PC?

The main program is written in python and for this reason, it is portable across different platforms. In principle, it is possible to run the code on Mac, Windows, and Linux, but we tested it only on Raspbian (Linux). It is important to note that, in the current state, the system uses a Raspberry Pi camera, a low cost, high-quality camera, designed specifically for Raspberry Pi. For this reason, changing the platform to Mac, Windows, or Linux would require to use a different USB camera and modify some of the code controlling the image acquisition accordingly. Please note that the Raspberry computer included in the system already outputs data and movies in formats (txt and avi) that can be read by any system.

I can see that video tracking can be a useful addition for welfare concerns to check the animals are ok following drug treatment for example but I’m struggling to see what other advantages it has (maybe in the situation where there is a depression of responding in the presence of a drug???).

We agree that video-tracking provides a useful addition for even more reasons than the one suggested by the referee: having a video stream of the mouse behavior provides useful information to the experimenter. If the experimental design includes drugs or manipulations possibly interfering with the animal’s welfare, visual inspection of the mouse behavior allows to reveal forms of discomfort, but also to control the experiment in real-time. For example, it is possible to stop a trial to avoid a button press during climbing or to discourage unwanted behaviors that risk being reinforced.

More importantly, The tracking is also crucial to broaden the applications of the system. Indeed, we also implemented a computer vision algorithm that performs real-time tracking opening two applications:

1) Tracking data can be exploited to analyze behavioral inhibition. Indeed, a new trial is triggered only if the animal stays for more than a user-defined period of time in the active area. A first bonus is that in this way, the position of the mouse at the beginning of each trial is known allowing precise measure. Second, the animal has to inhibit itself to exit the active area too early in order to activate the trial and get the reward . By progressively increasing the time required for the mouse to stay in the active area, it is possible to measure impulsivity or reproduce neuropsychological tests used on humans such as delayed gratification or stop signal task. We are exploiting this possibility in a mouse mutant line showing a strong reduction in the number of trials if the time required to remain in the active area is increased. Moreover, these impulsive mice also commit more false alarms (trying to perform a trial when it is not active) in triggering a new trial.

2) Moreover, we used spatial tracking to measure the total distance moved and the velocity of the animals and to examine exploratory patterns. For example, we are starting to apply the use of machine learning or other statistical procedures such as principal component analysis, to highlight subtle differences in strategies between different genotypes.

Detailled comments:

Abstract:

Line 9: ‘Fully 3D printable’ is a bit misleading.

There are many parts required to run the device that are not 3D printable - without these parts, the 3D box would be entirely non-functional. Here, I think just removing the word ‘fully’ would be sufficient.

We agree and removed the word fully according to the reviewer’s suggestion.

See also line 22 in significance statement ’only 3D printed parts’ - this should be toned down, perhaps ‘largely’.

We replaced the word “only” with “largely” as the reviewer suggested.

I would suggest removing the references to the price in the abstract and also the intro (i.e. 150 Euro).

This is of course very changeable and may not be accurate over time, and is also not very internationally relevant. Referring to the device as a low-cost option is sufficient, and the prices can be kept in Table 1 as a reference for specifics.

We agree with this comment and removed the references to price.

Introduction:

Line 38/39, the citation here of the Escobar 2015 study in relation to a statement about commercially available and expensive systems is a bit misleading as this is a paper describing a custom made low-cost operant training device. Either insert something like ‘except see...’ before this citation, move the citation to after ‘do not provide customizable features’ (if this was the point to be made with this citation), or reference other studies supporting the assertion.

We cited Escobar 2015 to support our statement about the expensiveness of other commercially available systems. To resolve the ambiguity we added the sentence “As previously suggested” and expanded the list of references endorsing this claim.

Methods:

In terms of methodology described:

Please state the number of hours (23) of water restriction explicitly in the methods. Also state when lights were on in the light cycle as well as the strain, sex, age and supplier of the mice.

We added these details in the Mice housing and handling section.

It should be stated for those that are not experts in the area that it is not common to test mice 3 times per day (because normally larger cohorts than 6 are used) so the learning curves may not be representative of those produced with daily training sessions.

We added consideration about this in the discussion section.

For the design of the chamber, it would be better to have the port on the opposite wall to the reward spout. This more optimal to prevent the mice from simply moving sideways from the response window to the food chamber which is easier.

We agree that it is very important to prevent the mice from simply moving sideways triggering multiple reward deliveries. Classically this is achieved by placing the reward spout on the opposite side of the buttons. In this OC chamber, we solved this problem using video tracking. In order to start a new trial it is required for the mouse to move into an active area located on the arena side opposite to the interface wall and remain there for a user-defined amount of time.

Is there a house light?

All of the experiments are carried out during the light phase in dimmed light conditions. However, the OC chamber can run both in complete darkness and in normal light conditions. The camera is equipped with

IR LEDs that provide sufficient illumination to perform tracking without the aid of external illumination.

Is there a removable tray for cleaning purposes? Mice do not like walking around in their own urine and faeces.

Since the box is joint assembled, the frontal interface panel can be easily removed for cleaning. We routinely clean the arena with 20% ethanol at the end of every session. We also designed a second version (included in the extended data, with holes on the floor). Moreover the entire box is coated with epoxy resin to ensure a proper cleaning of the surfaces.

Please indicate the version of Python used.

The script has been tested on Python 3, the LCD version of the code instead, runs on Python 2.7.

Line 75: the authors list their specific printer (Kentstrapper Verve 3D printer), but it would be very useful to know what are the important properties/parameters for 3D printing this specific operant device. Clearly, this is only a cheap alternative if labs already have a 3D printer equipped, so it would be useful to know explicitly if the properties of their model would be compatible with the design. Here, I would suggest listing some key parameters that are necessary for printing success with any model printer (minimum dimensions, resolution, filaments; any limitations?).

The design of the OC chamber is quite simple, the components have relatively big dimensions for

3D-printing and do not require a high degree of precision. For this reason, there are no known limitations since almost all the printers are sufficiently precise to successfully print the entire chamber. We used the slicer software Cura, and printed with a medium resolution (100 μm), printing speed is 90 mm/s, infill of 20%. The filament diameter is 1.75mm. We added these details in the readme file.

Line 86: a small statement about why graphene is used as the capacitive touch sensor (and any suggestions on alternatives that do/do not work that may have been tested) would be useful.

Any conductive material can in principle be used as a capacitive sensor, however, the main advantage of using conductive PLA resides in the possibility to build specific button designs to meet different experimental needs (e.g. motor impairment, nose poking, etc.).

Line 87: reference for conductive glue?

Added in the text https://www.chemtronics.com/circuitworks-conductive-epoxy-2

Line 84: camera reference? I assume Pi camera from table 1. Please include this here.

We added this reference in the text.

Line 96: what precision does the stepper motor plunger have for reward dispensing? What is the smallest/largest reward drop size, is this adjustable, etc.

The smallest drop size tested is 7,05 μL with a standard error of 0,78 μL. In our experience setting a smaller drop size results in unreliable delivery. The largest drop size is only limited by the viscosity of the reward solution. The amount of liquid is proportional to the rotational movement of the stepper motor. As commented in the code file, this quantity can be modulated by editing a low-level parameter in the file “stepper.ino” to adjust for one’s experimental needs.

Line 110: what are the properties of the acoustic stimulator (frequency range etc). (60dB and a frequency range of 2kHz to 10 kHz) https://www.adafruit.com/product/160

Line 141: what does a frame rate greater than 10Hz mean? 10 Hz is the minimum? What frame rate do the authors routinely use? What is the max the system (Pi camera, Pi interface) can handle?

Depending on the dimension of the frame acquired you can have a different framerate. We specified in the corrected text that in our experiments we used a framerate of 20 fps with a 200x200 pixels ROI.

Line 143: How is this GUI run/accessed with the available code (see also comments on extended data 2).

We added this information in the readme.md

Results:

In the extended data (movie 1), it is perhaps difficult to tell from the top view, but it appears as though the mouse sometimes touches the button on the correct side (sometimes climbing on top of the device, for instance at 3:30 in movie 1) but this does not set off the sensor until the mouse touches the front of the panel (?). Could the authors comment on this design, the apparent behaviour, and the sensitivity of the touch device in this regard. If it is the case that the front is more sensitive than the top/sides, would it not be more advantageous to put the touch sensors recessed into the frame of the device so that only the front surface is exposed to the animal. It would seem that this would produce more of a one-to-one relationship towards touching the correct button and receiving the reward. As it is, the mouse likely has some extinction because it is touching the correct side button but in the ‘wrong way’ so that does not set off the sensor. Can the authors comment on this either as an option or as a experimental design consideration. This would also clearly affect the validity of reaction time measures (could the authors comment on this in regard to long-term RT measures; Line 206). If it is the case that it is difficult to see from the video, and the touch sensor is activated from all sides, then please state this explicitly in the methods description and comment on the fact that the animal can develop many different strategies to activate the sensor (i.e. climb up on, hit side of, hit front of, etc), reducing the stereotyped nature of the behaviour and potentially affecting RT measures.

We agree with the referee that this is an important point. From the top view in the movie, it might seem that there could be a degree of unreliability in the touch buttons and this could result in some process of extinction. With regards to the sensitive area of the buttons, we confirm that they are entirely printed with an electrically conductive material (graphene-containing PLA) and for this reason, all the parts of the button are equally sensitive to the touch. The sensibility of the buttons is manually checked by the experimenter at the beginning of every experimental day, by setting a threshold that allows the buttons to set off by touching them extremely lightly with bare hands, thus we are confident that “false negative” errors are reduced as much as possible. We included the Arduino program that helps the experimenter to set a proper threshold for the touch buttons in the extended data folder in arduino_files/skinnerCapacitiveTest/skinnerCapacitiveTest.ino .

Unfortunately the top view in the movie1 is not ideal to check the mouse body position, but given the points mentioned above and our experience with the device we are confident that in the “failed” attempts, the mouse actually never touched the buttons with its paws but instead it stood on the lower border of the display. In fact, when the correct button press is detected, it is possible to see that the mouse is touching the left side of the right button with its paw (frame# 6425, time 03:34). We agree, however, that the fact that the buttons can be activated from all sides allows the animals to develop diverse strategies to touch the button. In our opinion, this feature could further help to differentiate between mice with different genotypes or treatments that might learn, prefer or use different strategies. Moreover, given that the entire button surface is active, it is possible to produce different designs of the buttons thus refining the affordance of the object for the mice. As requested we added the following sentence in the Results: “It is interesting to note that, since touch sensors are activated from all sides, some of the variability in timing performance could be explained by the development of different strategies to activate sensors.”

As far as we can tell, the extended data 2 does not include a readme file.

This should be standard and would help with installation as well as general description of the provided code and associated files. Additionally, without this, it is difficult to evaluate how user friendly the open source software is for this system. Either way, we would encourage the authors, either in a readme file in extended data 2 or in a supplementary information directly within the manuscript, to provide a small example of code needed to run the system. Such as a simplified demo bit of code to get users started with the basic functions. In our experience, without this users can very quickly become frustrated and will not pursue using the open source system.

We strongly agree with the reviewers that a readme file is essential to get users started with the apparatus and to increase its usability. During the first submission, we removed the readme.md file because it contained multiple references to personal repositories, thus infringing the double-blind policy of eNeuro review process. We now added the file. The file contains an introduction to the device, a description of the content of the repository and more detailed instructions on the few parts of the code that can be edited to customize the experiments or for testing purposes. The final version of the readme.md file will be analogous and will only contain a final section with contact information and links to personal repositories. Please note that the code files has also been more thoroughly commented.

Discussion:

We are missing a more detailed description of how the current results using this OC chamber relate to other standardized systems/publications.

Line 234: the authors state the results produced stereotyped learning curves that were reproducible across mice, which speaks to the internal validation of the OC chamber, but how do these learning curves relate to extensive previously published data on operant conditioning? This discussion would be important for external validation of this system.

We agree with the reviewer. We expanded the comments on internal/external validity in the discussion by adding a comparison with the most similar paradigm present in the literature that employs a commercial device from Med Associates ( Malkki et al. 2010 ). The values collected with this device are similar to those compared with our system for both nose pokes and lever presses.

It would also be helpful to expand on what the range of tasks is that can be performed with the current device being described.

We added a possible application of the chamber to animal models of disease in the discussion. Here we evaluate the possibility to test inhibitory control using video tracking: by increasing the time required to trigger a trial, it is possible to measure impulsivity or reproduce neuropsychological tests used on humans

Line 245-248: The Francis and Kanold, 2017 system is designed for mice, contrary to the authors claims that this system and others are designed for rats. This is also a highly automated system that can be used in a home cage without extensive researcher supervision. Further, what would be the rationale that the Escobar 2015 system could not be used with mice? In relation to this, we would generally like to see a more extensive comparison with previous devices/literature including specific advantages (and disadvantages) of the proposed system to aid in evaluation of the needs for specific experiments before implementation. What precisely does this system add to push the field forward. For instance, the combination of realtime tracking and analysis of movement together with the system is of interest and could be better highlighted in this regard (including references to previous designs).

We agree with the reviewer opinion about the systems for rats or mice, so we completely removed the claim. We changed the discussion focusing on other advantages of our system: “the main strength of the present device is the high degree of reproducibility, since the model is standardized and can be downloaded to print the same structure with the same materials throughout different laboratories.

Secondly, it can be customized in relation with specific experimental needs (e.g. scaling the size of the chamber to study very young animals). Lastly, different versions of the OC chamber can be tested and shared through on-line repositories, such as Thingiverse ( https://www.thingiverse.com/ ) and NIH Print

Exchange ( https://3dprint.nih.gov/ ). Moreover, the OC chamber includes real time tracking of the mouse position, a feature that could be used as second phenotyping measure of anxiety or stereotyped behaviors. Additionally it allows to analyse other aspects of behavior, such as inhibitory control (Munakata et al. 2011) . For example, by increasing the time required to trigger a trial, it is possible to measure impulsivity or reproduce neuropsychological tests used on humans like delayed gratification or stop signal tasks (Pinkston and Lamb 2011; Furlong et al. 2016) .”

The authors mention that the LED display can be ‘easily’ functionally expanded using Pygame to allow for more complex visual displays. We think this would be a significant advantage. Although this would increase the cost of the system, including this as an ‘option’ in the code and as an example would increase the appeal for this system. As is, we are not sure of the widespread usefulness for a simple left/right light device. If this is as easy as the authors suggest, we would like to see it as an option from the start, at least as a backbone for implementation in the open source code. In general, this functionality would significantly add to the novelty of this OC chamber.

We thank the reviewers for the comment. We implemented a backbone for functionality with a Raspberry Pi- compatible LCD screen (Kookye 3.5” for RPI3, ∼= 30€). We choose this model for two reasons: first, it is compatible with Raspberry Pi and, as a result, does not require the cost of an external PC to be added to the price of the setup; second, it is natively a touch screen display, and we think that developing a basic implementation for this device could help future users to expand the functionality of the OC chamber even more. Since the Raspberry Pi already uses Python to run the OC chamber code, we installed the python library PsychoPy, a widespread toolbox used for visual stimuli presentation in humans and animals. Since we implemented only an example visual stimulus, we choose to use

PsychoPy because it already has vast support material for users who want to code their customized visual stimuli (Extended reference manual, books, youtube tutorials, and workshops hosted by the

University of Nottingham, https://www.psychopy.org/resources/resources.html ). In the folder LCD_oc_chamber we added a version of the code that performs the same behavioral task but uses a sample image displayed in different portions of the screen instead of the LED lights. We also included a video showing this version of the chamber in extended_data_3, we mentioned this version in the README.md file, and we included a 3D project for a version of the interface wall that fits this LCD screen.

Line 255/256 and 260/261: if the authors would like to claim that this is a useful tool for training etc, then it is especially important to have well documented code (comments throughout the python code are generally few and some comments are not in English) and a clear readme file with installation instructions, demo code etc (see also point regarding extended data 2 above).

We agree with the reviewers. In order to make the code more user-friendly, we translated all the comments that were not in English. We also improved the quantity and quality of comments throughout the code.

For the few parts of the code that are intended to be edited by a user to customize the experiments, we tried to be as detailed as possible with the comments, we tried to make it editable even by a user with no experience whatsoever in programming and we also provided a short guide to which are the files that are supposed to be edited in the readme.md file. The low-level code that is supposed to be edited only by power users has been commented as well to give a sense of what the sections of the code are for, but we assumed that users that edit these portions already have experience in python programming. A well documented readme file has also been included.

Table 1: the authors list vendors for products as amazon, but of course amazon is only the distributor not the manufacturer of these products. Please list the manufactures name as well as a manufactures’ product code

where possible. In the future, amazon may stop supplying these brands and the information would be lost. In table 1, perhaps the authors could keep the amazon reference (depending on journal policy), but in the text and main reference for table 1 I think the manufactures’ data would be more useful.

We thank the reviewers for the comment. We edited the table to explicitly mention the manufacturer and the product code next to the distributor.

Minor comments:

Line 247: typo ‘has’ not ‘have’

We edited the phrase containing this word.

Line 248: typo ‘includes’; add s

We corrected the typo Table 1: add (Euro) after Price heading We added the currency symbol
