# Supplementary figures and images for: 3D Printable Device for Automated Operant Conditioning in the Mouse
Source: eNeuro. 2020 Apr 27;7(2):ENEURO.0502-19.2020. doi: 10.1523/ENEURO.0502-19.2020 (PMC7218003; doi:10.1523/ENEURO.0502-19.2020)

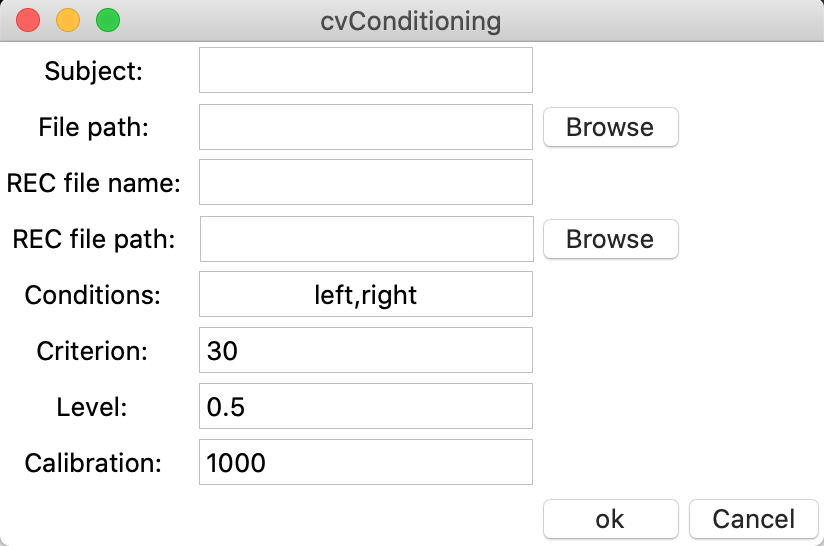

Supplement: Extended Data 1 — zip contains the code for both Arduino and Raspberry Pi boards. Download Extended Data 1, ZIP file. [file enu-eN-OTM-0502-19-s03.zip › EXTENDED_DATA_1/ gui_example.png]

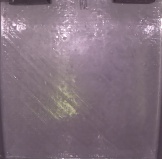

Supplement: Extended Data 1 — zip contains the code for both Arduino and Raspberry Pi boards. Download Extended Data 1, ZIP file. [file enu-eN-OTM-0502-19-s03.zip › EXTENDED_DATA_1/template.jpg]

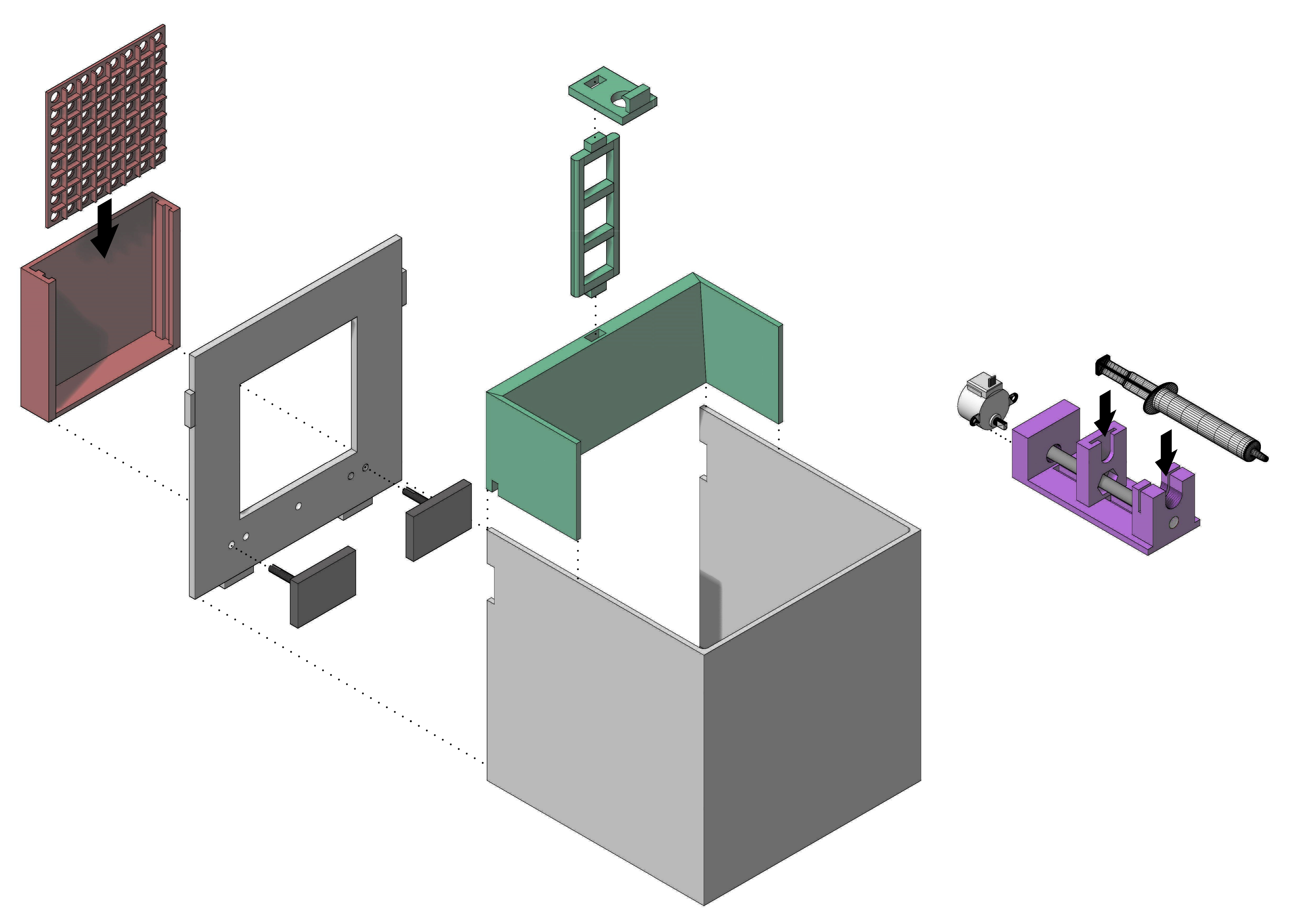

Supplement: Extended Data 1 — zip contains the code for both Arduino and Raspberry Pi boards. Download Extended Data 1, ZIP file. [file enu-eN-OTM-0502-19-s03.zip › EXTENDED_DATA_1/figures/EXPLODED VIEW.jpg]

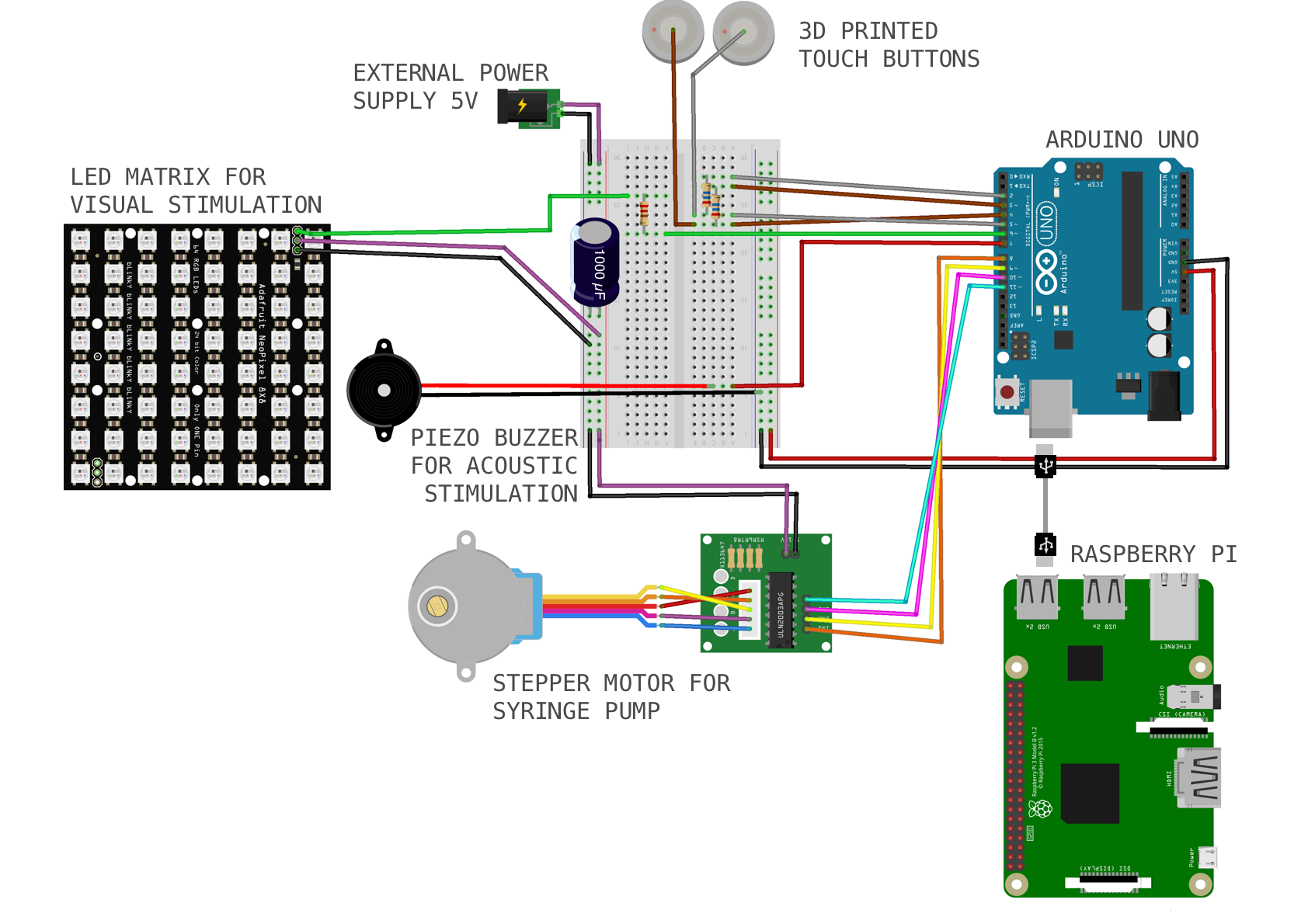

Supplement: Extended Data 1 — zip contains the code for both Arduino and Raspberry Pi boards. Download Extended Data 1, ZIP file. [file enu-eN-OTM-0502-19-s03.zip › EXTENDED_DATA_1/figures/diagram_scheme.png]

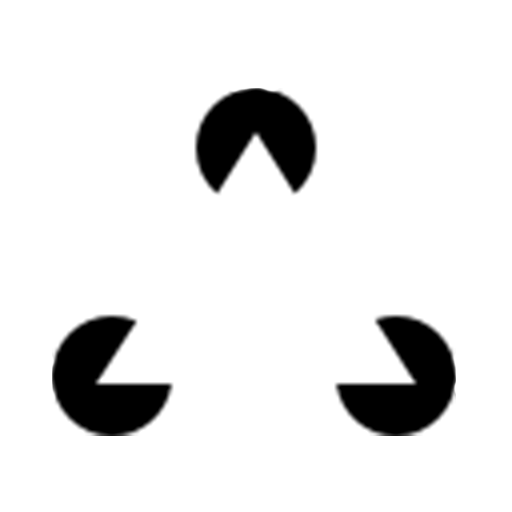

Supplement: Extended Data 1 — zip contains the code for both Arduino and Raspberry Pi boards. Download Extended Data 1, ZIP file. [file enu-eN-OTM-0502-19-s03.zip › EXTENDED_DATA_1/LCD_oc_chamber/test_images/iTriangle.png]

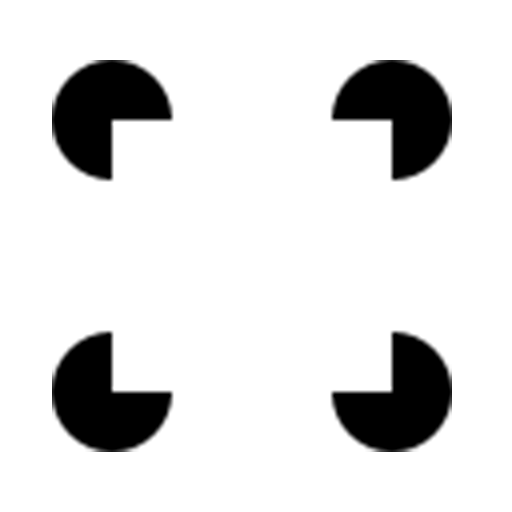

Supplement: Extended Data 1 — zip contains the code for both Arduino and Raspberry Pi boards. Download Extended Data 1, ZIP file. [file enu-eN-OTM-0502-19-s03.zip › EXTENDED_DATA_1/LCD_oc_chamber/test_images/iSquare.png]

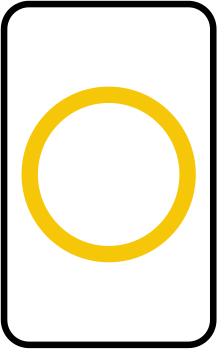

Supplement: Extended Data 1 — zip contains the code for both Arduino and Raspberry Pi boards. Download Extended Data 1, ZIP file. [file enu-eN-OTM-0502-19-s03.zip › EXTENDED_DATA_1/LCD_oc_chamber/zener/circle.png]

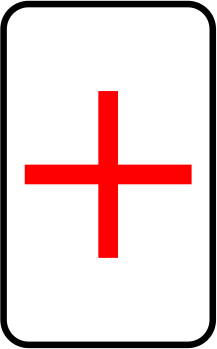

Supplement: Extended Data 1 — zip contains the code for both Arduino and Raspberry Pi boards. Download Extended Data 1, ZIP file. [file enu-eN-OTM-0502-19-s03.zip › EXTENDED_DATA_1/LCD_oc_chamber/zener/cross.png]

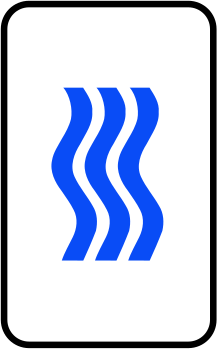

Supplement: Extended Data 1 — zip contains the code for both Arduino and Raspberry Pi boards. Download Extended Data 1, ZIP file. [file enu-eN-OTM-0502-19-s03.zip › EXTENDED_DATA_1/LCD_oc_chamber/zener/waves.png]

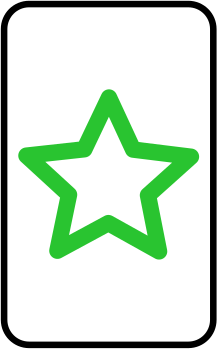

Supplement: Extended Data 1 — zip contains the code for both Arduino and Raspberry Pi boards. Download Extended Data 1, ZIP file. [file enu-eN-OTM-0502-19-s03.zip › EXTENDED_DATA_1/LCD_oc_chamber/zener/star.png]

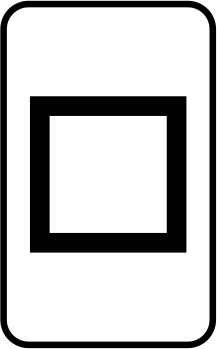

Supplement: Extended Data 1 — zip contains the code for both Arduino and Raspberry Pi boards. Download Extended Data 1, ZIP file. [file enu-eN-OTM-0502-19-s03.zip › EXTENDED_DATA_1/LCD_oc_chamber/zener/square.png]
